# Oral Hygiene Practices and Oral Health Knowledge among Students in Split, Croatia

**DOI:** 10.3390/healthcare10020406

**Published:** 2022-02-21

**Authors:** Antonija Tadin, Renata Poljak Guberina, Josipa Domazet, Lidia Gavic

**Affiliations:** 1 Department of Restorative Dental Medicine and Endodontics, School of Medicine, University of Split, 21000 Split, Croatia; josipadomazet97@gmail.com (J.D.); lgavic@mefst.hr (L.G.); 2 Department of Prosthetic Dental Medicine, School of Medicine, University of Split, 21000 Split, Croatia; poljak@sfzg.hr

**Keywords:** knowledge, oral health, oral hygiene, practice, students

## Abstract

Background: Knowledge of oral health is a fundamental prerequisite for healthy behavior, allowing individuals to take measures to protect their overall health. This cross-sectional study aimed to examine the knowledge of oral health as well as to assess the oral hygiene habits among healthcare and non-healthcare students. Methods: The study was based on a questionnaire and was conducted among 1088 students. Most of the students, 67.6% were non-healthcare students. Data were processed by Mann–Whitney or Kruskal–Wallis one-way ANOVA test. Results: The correct median score and interquartile range were 11 (9–13) for all surveyed students, 11 (9–12) for students in non-healthcare programs and 13 (11–14) for students in healthcare programs. Students did not significantly differ in the knowledge of oral health by gender (*p* = 0.082) but did differ by age, study program, and year of study (*p* ≤ 0.001). Students whose family members work in the field of dental medicine also showed better oral health knowledge (*p* ≤ 0.001). Conclusion: The results showed good oral health knowledge among tested university students. However, it is important to emphasize that students who showed better knowledge more often used additional aids to maintain oral hygiene; therefore, the obtained data underline the importance of students’ further education in order to better understand and maintain oral health.

## 1. Introduction 

There is much more to oral health than beautiful and healthy teeth. It is fundamental to overall health and affects the wellbeing and quality of life of every individual [1]. Oral health affects an individual’s oral functions and social interactions, and it is closely linked to overall health and quality of life [2,3,4].

Oral health is an integral part of overall health, and each influences the other [3,5,6,7]. Improper diet, smoking, alcohol intake, and poor oral hygiene practices are the most significant factors influencing the occurrence of various oral diseases. Diet affects the development of dental caries, dental erosion, periodontitis, oral cancer, and many other diseases of the soft tissues of the oral cavity. Smoking has been linked to oral cancer, gingival and periodontal disease, periimplantitis, tooth discoloration, halitosis, taste bud changes, and difficulty healing wounds after surgery. High alcohol intake is associated with an increased risk of developing oral cancer or other potentially malignant disorders, periodontitis, dental caries, and xerostomia. Poor oral hygiene can lead to the development of dental caries and periodontitis, and is also associated with heart disease, cancer, and diabetes [7,8,9,10,11]. 

Many of these oral diseases are preventable through education about risk factors. Oral hygiene is a critical factor in maintaining good oral health, and subsequently is related to overall health and quality of life. The most effective method for preventing dental caries or periodontitis is the removal of dental plaque by regular and proper mechanical cleaning of the teeth, a key step in maintaining oral health [4,12,13]. 

Knowledge of oral health is a fundamental prerequisite for healthy behavior, which allows an individual to take measures to protect their own health. Different researches have shown links between increased knowledge of oral health and better oral hygiene and health-related behaviors [4,14,15]. Many studies about oral hygiene practices and knowledge have been conducted among university students in different parts of the world, but this is the first such study conducted at the University of Split. This study aimed to examine and compare oral health knowledge among healthcare and non-healthcare students, and to assess their respective oral hygiene habits and students’ own self-assessments of their oral health. The goals of the study were to determine if there is a difference in oral health knowledge among respondents from different scientific fields, and if there is a difference in oral hygiene habits among respondents depending on their oral health knowledge.

## 2. Materials and Methods 

This cross-sectional survey, in the form of a questionnaire, was conducted during February and March of 2021 at the Department of Restorative Dental Medicine and Endodontics of the School of Medicine, University of Split, Croatia. The study was conducted following all ethical principles, including the Helsinki Declaration of the World Medical Association, and was approved by the Ethics Committee (Class: 003-08/21-03/0003, No.: 2181-198-03-04-21-0012). 

The online survey questionnaire, which was based on several surveys related to the same topic, consisted of four parts and contained 43 questions [14,16,17,18]. The first part included primary demographic and professional data (gender, age, study, year of study, employment of a family member in health/dental medicine, assessment of socioeconomic status) of respondents. The second part contained 15 closed questions related to knowledge about oral health and its maintenance. Each correct answer in the second part was scored with one point, and incorrect answers scored zero points, for a maximum possible score of 15. The sum of correct answers for each respondent was taken as a measure of their overall oral health knowledge, and was the primary result considered in the study’s analysis. The third part consisted of 11 questions related to the oral hygiene habits of the respondents (including frequency of brushing, use of fluoride toothpaste, duration of brushing, toothbrush hardness, brush type, frequency of changing toothbrush, brushing technique, use of dental floss, use of interdental brush, use of mouthwash, and tongue washing). The fourth part consisted of three questions related to the usage of dental services (frequency of visits to the dentist, the reason for the last visit, time since the last visit), six questions were relative to the self-assessment of oral health (including number of fillings, extracted teeth, number of endodontically treated teeth, bleeding gums, dental hypersensitivity, and bad breath) and one multiple-choice question about source of oral health information (dentist, school, family and friends, media).

Experts from various fields of dental medicine (specialists in pediatric and preventive dentistry and specialists in endodontics and restorative dentistry) endorsed the content of the prepared questionnaire. As a reliability test, the questionnaire was administered to 30 students (15 from healthcare and 15 from non-healthcare studies) who confirmed the comprehensibility of the survey questionnaire. These questionnaires were not included in the dataset of the primary study. The internal consistency of the questionnaire in the pre-testing phase was satisfying, with a Cronbach’s alpha coefficient of 0.71.

Students of seventeen different programs completed the questionnaire: four related to healthcare (Medicine, Dental Medicine, Pharmacy, and University Department of Health Studies) and thirteen unrelated to healthcare (Economics, Electrical Engineering, Mechanical Engineering and Naval Architecture, Philosophy, Civil Engineering, Architecture and Geodesy, Theology, Chemistry and Technology, Kinesiology, Maritime Studies, Law, Nature Science, Marine Studies, and Forensic Sciences) of the University of Split, Croatia. The study included 1088 adult students of both genders from all years of study who completed the questionnaire. The questionnaire was designed in the form of an online survey (Google Forms), and its link was sent to the student representatives of each program, who then forwarded it to other colleagues. The criteria for participation were students during the academic year 2020/2021, attending one of the programs at the University of Split, who fully completed the questionnaire. Minor students, and those who did not fully complete the questionnaire, were excluded from the study. The research objectives of the study were explained to all participants at the beginning of the questionnaire. Participation was entirely voluntary and anonymous. 

The minimum required sample size (*n* = 377) was calculated from the total number of students who studied at the University of Split in the academic year 2020/2021 (N = 18,026) with a 95% confidence interval, a 5% error limit, and a response distribution of 50% (Sample Size Calculator by Raosoft Inc., Seattle, WA, USA). 

The data were analyzed by the Statistical Package for Social Sciences (SPSS, IBM Corp, Armonk, New York, NY, USA) version 25. The Kolmogorov–Smirnov test was used to assess the normality of the distribution of responses. Descriptive analysis calculated the frequency and percentage of categorical data, and quantitative data were expressed as the median and interquartile range (IQR). Statistical analysis was performed using the Mann–Whitney or Kruskal–Wallis one-way ANOVA test. The significance level was set at *p* < 0.05. 

## 3. Results 

Table 1 presents the sociodemographic characteristics of the respondents; 1088 students participated in the study, of which 869 (79.9%) were women. The mean age of the subjects was 22.91 ± 2.62 (min 18, max 39). Most respondents (N = 352; 32.4%) attended one of the programs in healthcare (medicine, dentistry, pharmacy, or health studies). Respondents did not significantly differ in their knowledge of oral health by gender (*p* = 0.082), but did differ by age, program, and year of study (*p* ≤ 0.001). Students whose family members work in dental medicine also showed better knowledge of oral health (*p* ≤ 0.001). 

Table 2 shows the frequency of correct and incorrect answers to the questions regarding oral health; 13 of the 15 questions were answered correctly by most respondents; 14 (1.3%) of the respondents did not answer any questions correctly, while 47 (4.3%) answered all questions correctly. The median correct score was 11 (9–13) for all surveyed respondents; 11 (9–12) for respondents in non-healthcare programs, and 13 (11–14) for respondents in healthcare programs. 

Table 3 and Table 4 show oral hygiene habits among the respondents. Most respondents brush their teeth with a toothbrush and toothpaste several times a day (85.7%), with over 60.1% of respondents using fluoride toothpaste. Interestingly, 13.6% of respondents do not use fluoride toothpaste, 38.6% never or rarely use dental floss as an aid to maintain oral hygiene, and 65.1% never or rarely use interdental brushes. Only 26.7% and 15.3% of respondents use dental floss and interdental brushes daily, 59.5% of the respondents report daily tongue washing, and only 20.9% use mouthwash daily. Most respondents change their toothbrushes every three months. About 70% of respondents brush their teeth for two to three minutes; only 2.5% do so for more than five minutes. Better knowledge was shown by subjects who use dental floss two or more times a day when compared to those who use it rarely or not at all (*p* = 0.018; *p* = 0.016). This was also the case when comparing those who regularly use interdental toothbrushes to those who do not use them, or use them infrequently (*p* = 0.038; *p* = 0.048). Interestingly, poorer knowledge was shown by subjects who do not use fluoride toothpaste (*p* ≤ 0.001) compared to those who use them. 

Table 5 shows the frequency and reasons for using dental services, as well as self-assessment of oral health by respondents. More than half of the respondents (55.5%) visited their dentist within the last six months; 59.9% of respondents stated that their most recent visit to a dentist was a regular check-up, while 32.9% stated that a problem with a tooth or orofacial structures motivated their most recent visit. Respondents who reported the former scored higher on oral health knowledge that those who reported the latter (*p* ≤ 0.001). The average number of fillings in respondents was 4.00 ± 3.17 (min 9, max 19), the average number of extracted teeth was 0.36 ± 0.84 (min 0, max 7), and the average number of endodontically treated teeth was 0.61 ± 1.14 (min 0, max 8). In addition, about three-quarters of the subjects reported experiencing bad breath, tooth hypersensitivity, and/or bleeding from the gums.

Dentists were the most common (80.6%) source of oral health information followed by parents (33.1%), family and friends (70.4%), media (55.9%) and school (33.7%).

## 4. Discussion 

This study aimed to assess knowledge of oral health, oral hygiene habits, and self-assessment of oral health among students at the University of Split. Knowledge of oral health enables the achievement of a high standard of oral health and related tissues. It is a crucial prerequisite for responsible behavior towards one’s health. 

The results of a survey of 1088 students at the University of Split found significant differences in knowledge about oral health among respondents from different scientific fields (*p* ≤ 0.001). The median response to oral health knowledge questions was 11 (9–13) for all respondents, while it was 11 (9–12) for respondents in non-healthcare programs, compared to 13 (11–14) for those in healthcare programs. Research on the student population of Saudi Arabia has also shown that the level of knowledge about oral health is lower in non-medical colleges than it is in medical colleges [14]. It has also been shown that a higher level of knowledge is positively correlated with age (>22 years versus ≤22 years) [14], and this was confirmed by this study, as we found that at the University of Split, older students (≥23 years) show better knowledge of oral health than younger students (*p* ≤ 0.001). 

In this study, respondents did not show a difference in knowledge depending on gender (*p* = 0.082), which is consistent with results of research on postgraduate students in India [16]. In contrast, the results of research conducted in Saudi Arabia [14] showed that female students have significantly better knowledge, attitudes and behaviors toward their oral health than their male counterparts. The positive oral health behaviors and attitudes of women in the study, as mentioned earlier, could be explained by the generally greater concern about appearance in women. For this reason, women are more likely to visit a dentist and to educate themselves about oral health. 

Periodic dental examinations are important in preventing oral diseases, educating patients, and encouraging the maintenance of good oral hygiene [14]. Research on students at the University of Split showed that almost half (47.7%) indicated that the socio-economic condition of their family was above average, according to self-assessment. A study conducted in China in 2019 on a sample of 263 middle-aged respondents found a significant link between age, low educational level, and poor oral health. This also affected oral health knowledge, with respondents of lower socioeconomic status showing a lower level of oral health knowledge. Poor knowledge of oral health is associated with poor oral hygiene and a higher number of lost teeth [19]. It is known that quality of life is generally related to the socioeconomic status, as good material conditions facilitate access to goods and services, including oral health care [1]. 

The great majority of respondents correctly answered the statements “Poor oral hygiene can lead to the development of dental caries and periodontitis”, “oral health is closely related to the general health of the individual”, and “proper oral hygiene can prevent dental caries and periodontitis” (96.0%, 92.5%, and 91.8%, respectively). Research on students from various studies in Saudi Arabia showed that most participants (94%) of both sexes agreed that brushing their teeth prevented periodontal disease. However, a large proportion of both sexes did not show an understanding of the relationship between oral disease and systemic health problems [14]. We also found that respondents whose family members work in dental medicine showed better knowledge of oral health (*p* = 0.043) than others, which is probably due to the greater availability of information in the home environment. 

We found differences in oral hygiene habits among the respondents depending on oral health knowledge, except for duration of brushing (*p* < 0.159), the hardness of the toothbrush (*p* < 0.104) and toothbrush replacement interval (*p* < 0.349). When asked how many times a day they brush their teeth, most respondents answered that they brush their teeth with a toothbrush and toothpaste several times a day (85.7%) for two to three minutes (70.5%), which agrees with a survey conducted at a military college in Bucharest, which showed that the majority of respondents (78.3%) brushed their teeth twice a day (morning and evening), and more than half of the respondents spent three minutes on oral hygiene [17]. In a study by Peltzer and Pengpid [18] on a sample of 19,560 undergraduate students from 27 universities in 26 countries in Asia, Africa, and America, the results showed that 67.2% of students brush their teeth twice or more times a day, 28.8% approximately once a day, and 4.0% never. The prevalence of brushing teeth less than twice a day appears to be higher among students in low- and middle-income countries than in high-income countries; e.g., 52.2% in India, 35% in Lebanon, 32% in Turkey compared with 7.9% in Italy, or 25% in the United States. A survey of the student population in Zagreb showed that 83% of students brush their teeth two to three times a day, and 17% said they brush their teeth more than three times a day, all of whom were students at the Faculty of Dentistry [20]. A similar statement was made by students from Bjelovar, Croatia [21].

As for type of toothbrush used, the majority of our respondents (90.1%) use a hand-held toothbrush. Similarly, a survey in Bucharest found that 77.5% of respondents used this same type of toothbrush [17]. A study of the student population in Saudi Arabia found that only 9.4% of women and 13% of men used electric toothbrushes [14]. 

More than half of our respondents (59.7%) change their toothbrush every three months, and a relatively high percentage (24.5%) even more often once a month. Most respondents (53.8%) of a survey of the Military College in Bucharest change their brush every three months, and 34.3% once a month [17], while in Zagreb, 48.3% of students use the same brush for less than three months [22]. 

Furthermore, dental floss is used daily by only 26.7% of our respondents, and interdental brushes by only 15.3%. Students who used dental floss more often showed better knowledge than those who did not use it or used it very rarely. In addition, students who used interdental brushes regularly showed better knowledge than those who did not use them. Some previous research shows that participants who brush their teeth frequently and use dental floss have better oral health knowledge [14]; 74.8% of students in Zagreb use additional means for maintaining oral hygiene (dental floss, interdental brushes, or mouthwash) [22]. 

When asked about the frequency of visits to the dentist, slightly less than half of our respondents reported going to the dentist as needed (43.2%), while only 26.6% go to the dentist every six months. Contrast this with a study from Bucharest, which found that 35.8% underwent dental examinations twice a year, 29.1% once a year, and more than a fifth (22.5%) visited dentists only when they have toothaches [17]. A survey of the student population in Zagreb showed that 28% of respondents went to the dentist every six months (most of them were dental students), and the rest of the respondents went only as needed [20]. In a study by Peltzer et al. [18], 16.3% of students went to the dentist twice a year, 25.6% once a year, 33.9% rarely, and 24.3% never. Our survey showed that almost a third of respondents (32.9%) last visited a dentist due to tooth or orofacial structures (pain, swelling). However, as many as 59.8% of respondents cited regular check-ups as a reason, slightly higher than the European average of 50% [23]. 

Most respondents (73.6%) did not know how an avulsed permanent tooth from the mouth could be returned to the oral cavity due to dental trauma. Yet this is an urgent therapeutic procedure, performed at the accident scene, and it is very important for the outcome of treatment, its total cost, and the consequences for the child who experienced the accident [24]. Similar results were shown by a study where only 46.7% of medical students, 35.8% of the Faculty of Kinesiology, 22.3% of Early and Preschool Education, and 21.1% of Teacher Education knew the same [24]. Most of the respondents had bleeding gums, bad breath, and/or tooth hypersensitivity (74.6%, 74.1% and 78.6%).

Although oral diseases can be prevented, they tend to grow despite efforts to preserve oral health both in Croatia and in the rest of the world [3,4]. For better prevention, the existing levels of dental care and educational mechanisms should be harmonized and specific mandatory measures with possible sanctions introduced [25]. Standards and methods for promoting and achieving good oral health of children have significantly advanced globally in the last 20–30 years [26]. In today’s population of the Republic of Croatia, on the other hand, there is a lack of action on preventive dental care in that same period. Given the lasting consequences of dental caries and periodontal disease, and the costs that are then unavoidable and borne either by the state and/or the patient personally, it is justified to ensure preventive measures at every possible level [27]. 

The present study showed that dentists (80.6%) are the most common source of oral health information. These findings agree with previously published studies conducted on school children and university students [28,29].

This study has several limitations. First, it was cross-sectional, so no causal conclusions can be drawn. Second, this investigation was conducted with students at only one University, and the involvement of others could have yielded different results. Third, university students are not representative of young adults in general, and levels of oral health behavior, as well as socioeconomic and health risk behavioral variables may differ in other sectors of the population. A further limitation of the study was that all data collected were based on self-assessment, while a dental examination could objectively assess an individual’s oral health. In addition, the higher proportion of females and the healthcare students in the total number of respondents suggest an overall response bias for the survey. However, the probable reason for the better response of these students is a greater interest in oral health and health in general.

The obtained data from this study can serve the educational institutions to understand student knowledge better and, consequently, better promote student education through various lectures or workshops. It is certainly recommended that the research be conducted on student populations throughout Croatia. Furthermore, to conduct a dental examination in parallel to objectively assess the oral cavity’s condition.

## 5. Conclusions

In general, the results showed good oral health knowledge among university students. However, it is important to emphasize that healthcare students and those whose family members work in the field of dental medicine have shown better knowledge. It was also confirmed that students with a higher knowledge score use oral hygiene aids more frequently like dental floss, interdental brushes, and mouth rinses. From all the above, it can be concluded that education on this topic is fundamental for understanding and maintaining of oral health.

## Figures and Tables

**Table 1:** Demographic characteristics of students according to the average assessment of knowledge about oral health.

Characteristic		Frequency N (%)	Knowledge Median (IQR)	*p*
Gender	Woman	869 (79.9)	11 (9–13)	0.082
	Man	219 (20.1)	11 (9–13)	
Age (years)	18–22	500 (46.0)	11 (9–12.75) ^a,b^	≤0.001
	23–25	451 (41.5)	12 (10–14) ^a^	
	≥25	137 (12.6)	11 (10–14) ^b^	
Field of study	Biomedicine and health	352 (32.4)	13 (11–14) ^c,d,e,f^	≤0.001
	Social Sciences	188 (17.3)	11 (9–12) ^c^	
	Technical sciences	214 (19.7)	10 (8–12) ^d^	
	Humanities	133 (12.2)	10 (8–12) ^e^	
	Natural Sciences	201 (18.5)	11 (9–13) ^f^	
Year of study	1st year	285 (26.2)	11 (9–12) ^g,h,i^	≤0.001
	2nd year	187 (17.2)	11 (9–13) ^j,k,l^	
	3rd year	199 (18.3)	11 (9–12) ^m,n,o^	
	4th year	150 (13.8)	12 (10–13,25) ^g,j,m,p^	
	5th year	193 (17.7)	11 (10–14) ^h,k,n,r^	
	6th year	74 (6.8)	14 (13–14,25) ^i,l,o,p,r^	
Family members-healthcare employees	No	815 (74.9)	11 (9–13)	0.067
	Yes	273 (25.1)	12 (10–13)	
Family members-employees in dental medicine	No	998 (91.7)	11 (9–13)	0.043
	Yes	90 (8.3)	12 (10–13)	
Family financial status	Below average	104 (9.6)	11 (9–13)	0.013
	Average	574 (52.8)	11 (9–13) ^q^	
	Above average	410 (47.7)	12 (10–13) ^q^	

Data are presented as median (IQR) or as numbers (percentages). Statistical significance was examined by Mann–Whitney or Kruskal–Wallis one-way ANOVA test. A different letter in the superscript indicates a statistical difference between the groups (^a, c, d, e, f, i, l, m, n, o, p, r^ *p* ≤ 0.001, ^b^ *p* = 0.030, ^g^ *p* = 0.012, ^h,j^ *p* = 0.002, ^k^ *p* = 0.003, ^q^ *p* = 0.013). Statistical significance was set at *p* < 0.05.

**Table 2:** The frequency distribution (%) of students’ answers about oral health.

Question	Answer	N (%)
Oral health is closely related to an individual’s general health.	*Yes*	1006 (92.5)
	No	18 (1.7)
	I do not know	64 (5.9)
Certain systemic diseases can manifest in the oral cavity.	*Yes*	933 (85.8)
	No	8 (0.7)
	I do not know	147 (13.5)
Oral health is closely related to an individual’s quality of life.	*Yes*	958 (88.1)
	No	42 (3.9)
	I do not know	88 (8.1)
The most common oral diseases are dental dental caries, periodontitis and oral cancer.	*Yes*	854 (78.5)
	No	25 (2.3)
	I do not know	209 (19.2)
Poor oral hygiene can lead to the development of dental caries and periodontitis.	*Yes*	1045 (96.0)
	No	9 (0.8)
	I do not know	34 (3.1)
Diet affects the development of dental caries, periodontitis and oral cancer.	*Yes*	948 (87.1)
	No	33 (3.0)
	I do not know	107 (9.8)
Smoking is associated with the occurrence of oral cancer and periodontitis.	*Yes*	898 (82.5)
	No	22 (2.0)
	I do not know	168 (15.4)
High alcohol intake is associated with an increased risk of developing oral cancer, periodontitis and dental caries.	*Yes*	623 (57.3)
	No	53 (4.9)
	I do not know	412 (37.9)
The hygiene and health of deciduous teeth are just as important as permanent dentition.	*Yes*	851 (78.2)
	No	41 (3.8)
	I do not know	196 (18.0)
Proper oral hygiene can prevent dental caries and periodontitis.	*Yes*	999 (91.8)
	No	24 (2.2)
	I do not know	65 (6.0)
Fluorides have a protective role in the development of dental caries.	*Yes*	660 (60.7)
	No	30 (2.8)
	I do not know	398 (36.6)
Mouthguards can prevent sports-related injuries to the teeth and soft tissues.	*Yes*	816 (75.0)
	No	21 (1.9)
	I do not know	251 (23.1)
A permanent tooth avulsed from the mouth due to dental trauma can be returned to the oral cavity.	*Yes*	287 (26.4)
	No	260 (23.9)
	I do not know	541 (49.7)
Sports drinks and energy drinks can damage the tooth surface and cause erosion.	*Yes*	662 (60.8)
	No	37 (3.4)
	I do not know	389 (35.8)
Loss of teeth due to ageing is a physiological phenomenon that is not possible to prevent.	Yes	349 (32.1)
	*No*	273 (25.1)
	I do not know	466 (42.8)

Data are presented as whole numbers and percentages. Correct answers are italicized.

**Table 3:** Oral-hygienic habits—tooth brushing of students according to the average assessment of knowledge about oral health.

Question	Answer	N (%)	Knowledge Median (IQR)	*p*
How often do you brush your teeth with a toothbrush and toothpaste	Rarely	2 (0.2)	12.50 (11–12.50)	0.010
	Several times a month (2–3x)	43 (4.0)	11 (7–12) ^a^	
	Once a week	1 (0.1)	10 (10)	
	Several times a week (2–3x)	11 (1.0)	8 (5–14)	
	Once a day	99 (9.1)	11 (8–13)	
	Several times a day	932 (85.7)	11 (9–13) ^a^	
How often do you use fluoride toothpaste	Never	148 (13.6)	10 (7–12) ^b,c,d,e,f^	≤0.001
	Rarely	127 (11.7)	11 (9–12) ^b,g^	
	Several times a month (2–3x)	58 (5.3)	11 (9.75–12.75) ^c^	
	Once a week	36 (3.3)	11 (9–12.75) ^d,h^	
	Several times a week (2–3x)	55 (5.1)	11 (10–13) ^i^	
	Once a day	117 (10.8)	11 (8.50–13) ^e,j^	
	Several times a day	547 (50.3)	12 (10–14) ^f,g,h,i,j^	
How long do you brush your teeth	Less than a minute	97 (8.9)	11 (9–13)	0.159
	Two to three minutes	767 (70.5)	10 (8–13)	
	Three to five minutes	197 (18.1)	11 (9–13)	
	More than five minutes	27 (2.5)	11 (9–13)	
Toothbrush-hardness you use	Very soft “extra-soft”	161 (14.8)	12 (10–13)	0.104
	Soft	334 (30.7)	12 (10–13)	
	Medium	436 (40.1)	11 (9–13)	
	Hard	43 (4.0)	11 (9–13)	
	I do not know	114 (10.5)	10 (8–12)	
The sort of toothbrush-you use	Manual	980 (90.1)	11 (9–13) ^k^	0.002
	Electrical mechanical	69 (6.3)	12 (9–14)	
	Electric sonic	32 (2.9)	13.50 (12–14) ^k^	
	Electric ionic	7 (0.6)	9 (5–15)	
How often do you change your toothbrush	Once a month	267 (24.5)	11 (10–13)	0.349
	Every 3 months	650 (59.7)	11 (9–13)	
	Every 6 months	138 (12.7)	11 (9–13)	
	Every 12 months	32 (2.9)	10 (8–12)	
What brushing technique you use	Horizontal	37 (3.4)	11 (9–13.50) ^l^	0.002
	Vertical	8 (0.7)	14 (13–14) ^l,m^	
	Circular	214 (19.7)	12 (9.75–14) ^n^	
	Combination	829 (76.1)	11 (9–13) ^m,n^	

Data are presented as median (interquartile range (IQR) or as numbers (percentages). Statistical significance was examined by Mann-Whitney or Kruskal–Wallis one-way ANOVA test. The same letter in the superscript indicates a statistical difference between the groups (^a^ *p* = 0.015, ^b^ *p* = 0.032, ^c^ *p* = 0.029, ^d,m^ *p* = 0.012, ^e^ *p* = 0.004, ^f,g,j^ *p* ≤ 0.001, ^h^ *p* = 0.022, ^i^ *p* = 0.044, ^k^ *p* = 0.005, ^l^ *p* = 0.027, ^n^ *p* = 0.020). Statistical significance was set at *p* < 0.05.

**Table 4:** Oral-hygienic habits–oral hygiene aids of students according to the average assessment of knowledge about oral health.

Question	Answer	N (%)	Knowledge Median (IQR)	*p*
How often do you use dental floss	Never	192 (17.6)	11 (9–13) ^a^	≤0.001
	Rarely	229 (21.0)	11 (9–13) ^b^	
	Several times a month (2–3x)	128 (11.8)	11 (10–13)	
	Once a week	82 (7.5)	11 (8.75–13)	
	Several times a week (2–3x)	167 (15.3)	11 (8.75–13)	
	Once a day	187 (17.2)	11 (8.75–13)	
	Several times a day	103 (9.5)	12 (10–14) ^a,b^	
How often do you use interdental brushes	Never	455 (41.8)	11 (9–13) ^c,d^	0.003
	Rarely	254 (23.3)	11 (9–13)	
	Several times a month (2–3x)	82 (7.5)	12 (9–13)	
	Once a week	51 (4.7)	12 (10–13)	
	Several times a week (2–3x)	79 (7.3)	12 (9–14)	
	Once a day	81 (7.4)	12 (10–14) ^c^	
	Several times a day	86 (7.9)	12 (10–14) ^d^	
How often do you use mouth rinse	Never	248 (22.8)	11 (9–13) ^e^	0.002
	Rarely	283 (26.0)	11 (9–13)	
	Several times a month (2–3x)	123 (11.3)	12 (9–13)	
	Once a week	74 (6.8)	11 (10–13)	
	Several times a week (2–3x)	132 (12.1)	12 (10–14) ^e^	
	Once a day	135 (12.4)	11 (9–13)	
	Several times a day	93 (8.5)	12 (10–14)	
How often do you brush your tongue	Never	67 (6.2)	11 (9–13)	0.002
	Rarely	154 (14.2)	11 (8–13) ^f^	
	Several times a month (2–3x)	86 (7.9)	11 (10–13)	
	Once a week	52 (4.8)	10 (9–12)	
	Several times a week (2–3x)	82 (7.5)	11 (8–13)	
	Once a day	209 (19.2)	11 (9–13)	
	Several times a day	438 (40.3)	12 (10–13) ^f^	

Data are presented as median (interquartile range (IQR) and as numbers (percentages). Statistical significance was examined by Mann–Whitney or Kruskal–Wallis one-way ANOVA test. The same letter in the superscript indicates a statistical difference between the groups (^a^ *p* = 0.016, ^b^ *p* = 0.018, ^c^ *p* = 0.038, ^d^ *p* = 0.048, ^f^ *p* = 0.023). Statistical significance was set at *p* < 0.05.

**Table 5:** Use of dental services and self-assessment of oral health among students according to the average assessment of knowledge about oral health.

Question	Answer	N (%)	Knowledge Median (IQR)	*p*
Frequency of visits to the dentist	If necessary	470 (43.2)	10 (8.50–13)	0.104
	Every 6 months	289 (26.6)	11 (9–13)	
	Every 12 months	211 (19.4)	11 (10–13)	
	Once every few years	103 (9.5)	11 (10–13)	
	I’m not going	15 (1.4)	11 (8–13)	
The reason for the last visit to the dentist	Problem with tooth or orofacial structures (pain, swelling)	357 (32.9)	11 (9–13) ^a,b^	≤0.001
	Continuation of regular treatment	77 (7.1)	12 (10–14) ^a^	
	Regular check-ups	652 (59.8)	11 (10–13) ^b^	
	Never	2 (0.2)	14 (14)	
Time of last visit to the dentist	Within the last six months	604 (55.5)	12 (10–13)	0.097
	Between six and 12 months	203 (18.7)	11 (9–13)	
	More than a year ago	170 (15.6)	11 (9–13)	
	More than two to six years ago	109 (10.1)	10 (8–13)	
	Never	2 (0.2)	12 (12)	
Number of fillings (self-assessment)	0	152 (14.0)	11 (9–13)	0.887
	1–3	339 (31.2)	11 (9–13)	
	>3	597 (54.9)	11 (9–13)	
Number of extracted teeth (self-assessment)	0	862 (79.2)	11 (9–13)	0.699
	1–3	211 (19.4)	11 (9–13)	
	>3	15 (1.4)	12 (8–14)	
Number of endodontically treated teeth (self-assessment)	0	741 (68.1)	11 (9–13)	0.474
	1–3	300 (27.6)	11 (9–13.75)	
	>3	47 (4.3)	11 (8–13)	
Have you ever had bleeding gums?	No	276 (25.4)	11 (9–13)	0.504
	Yes	812 (74.6)	11 (9–13)	
Have you ever smelled an unpleasant breath from your mouth?	No	282 (25.9)	12 (9–13)	0.081
	Yes	806 (74.1)	11 (9–13)	
Have you ever felt tooth hypersensitivity?	No	233 (21.4)	11 (9–13)	0.494
	Yes	855 (78.6)	11 (9–13)	

Data are presented as median (interquartile range (IQR) or as numbers (percentages). Statistical significance was examined by Mann–Whitney, Kruskal–Wallis or one-way ANOVA tests. The same letter in the superscript indicates a statistical difference between the groups (^a^ *p* = 0.006, ^b^ *p* = 0.040). Statistical significance was set at *p* < 0.05.

## Data Availability

The data that support the findings of this study are available upon request from the authors.
